# Evaluating Parametric Form-Based Code for Sustainable Development of Urban Communities and Neighborhoods

**DOI:** 10.3390/ijerph19127389

**Published:** 2022-06-16

**Authors:** Yingyi Zhang

**Affiliations:** School of Architecture and Urban Planning, Beijing University of Civil Engineering and Architecture, Beijing 100044, China; zhangyingyi@bucea.edu.cn; Tel.: +86-159-2179-3823

**Keywords:** parametric Form-Based Code, sustainable development, LEED-ND, communities and neighborhoods

## Abstract

Parametric techniques have been implemented for planning projects in urban communities and neighborhoods. Form-Based Code, a representative planning approach, uses parametric techniques towards an efficient planning process with three-dimensional visualized schemes. However, the extent to which the parametric Form-Based Code integrates the sustainable development criteria is still unclear. This paper targets to evaluate parametric Form-Based Code towards sustainable development of urban communities. Methods of Leadership in Energy and Environmental Design for Neighborhood Development (LEED-ND) are hired. Criteria that related to health environment and sustainable development in LEED-ND, including smart location & linkage, neighborhood pattern & design, and green infrastructure & buildings work to test parametric Form-Based Code. Results indicate that parametric Form-Based Code are concordant with a small number of the criteria of smart location & linkage and green infrastructure & buildings. Criteria of neighborhood pattern & design are more moderately or strongly reflected in parametric Form-Based Code. Conclusions include criticism and valuable insights for the enhancement of parametric Form-Based Code towards healthy socio-environment and sustainable development of urban communities and neighborhoods.

## 1. Introduction

Form-Based Code is a urban planning approach of New Urbanism that adopted in several cities and counties in last decades. It is designed to facilitate compact development and mixed uses, encourage mixed-income communities, and generally make cities more sustainable [1]. According to Parolek’s research, Form-Based Code can work to create or recreate a specific urban morphology primarily by controlling physical form through city or country regulation [2]. It fosters predictable built results and a quality public realm by using form as the organizing principle for the code [3]. Recently, parametric techniques support Form-Based Code as they provide effective and dynamic design process. As a main component of digital methodology, parametric techniques base on algorithmic thinking that enable the expression of parameters and rules that, together, define, encode and clarify the relationship between design intent and design response [4,5]. In fact, parametric techniques have been broadly implemented in architecture, engineering and construction industries since last century. Nagy indicates that from the first experiment using parametric tools in architecture, it has become clear that these tools could bring similar benefits to urban projects, effective even in higher scale urban cases [6]. 

LEED-ND criteria is used in this paper to score the parametric Form-Based Code to demonstrate if it support urban communities’ sustainable development. LEED, abbreviation of Leadership in Energy and Environmental Design, is an American rating system developed through the collaboration of the United States Green Building Council, the Congress for the New Urbanism and the Natural Resources Defenses Council [7]. Although initiated in the USA, LEED now establish its presence globally; providing internationally adopted sustainable design, construction and operational guidelines and standards and benchmarks for a wider scope of project sectors [8]. For instance, the system covers a wide range of projects in Asia [9], and cooperates with the local rating criteria of Asian countries such as China, Japan and Singapore. LEED-ND, called LEED for a Neighborhood Development, works as a specific rating system to help planners and developers create sustainable communities and neighborhoods that not only protect the environment but also improve the quality of life of the whole area [10]. 

This paper focuses on the evaluation of parametric Form-Based Code towards sustainable development of urban communities and neighborhoods. It has two major goals: (i) an analysis of parametric Form-Based Code to demonstrate if it reflects the criteria of LEED-ND; (ii) an enhancement of parametric Form-Based Code in the aspects of smart location & linkage, green infrastructure & buildings, and neighborhood pattern & design. The research question askes: How to evaluate and enhance parametric Form-Based Code by using LEED-ND criteria for sustainable development of urban communities and neighborhoods? As a result of this analysis, strategies are proposed for creating more sustainable parametric Form-Based Code. The contributions of this paper will be, firstly, a further implementation of LEED-ND in the field of Form-Based Code. And secondly, an exploration of enhancing sustainability through measuring parametric planning in urban scale. Planners, urban designers and environmentalists can benefit from the findings when analyzing the extent to which certain planning and LEED-ND criteria should be considered towards a sustainable human settlement.

## 2. Literature Review

Many researchers, urban planners, technicians and implementers explored parametric Form-Based Code to improve urban planning towards a sustainable socio-environment [11,12,13]. Throughout history, Sulaiman and Almahmood argued in 2021, planning “codes” have been a key tool for regulating the built environment, while also being a reflection of socio-cultural values [14]. The researches about parametric Form-Based Code generally contains three aspects. 

First is implementing parametric technology in Form-Based Code. For instance, Kim and Clayton tested the use of parametric techniques to support Form-Based Code of Dallas, Texas, US [15,16]. They demonstrated that parametric techniques provided relatively accurate data and model comparing with Geographic Information System (GIS) approaches [16]. Besides, Athas and Fuadyah studied the parametric models of Form-Based Code in Bandung, Indonesia to assist planning in urban scale by using parameters or variables to manipulate the planning layouts [17]. The research indicates that parameters and variables help provide rational procedures for regulating urban morphology. In Kim, Bimal and Jayedi’s opinion in 2020, parametric method improves stakeholders’ understanding of how Form-Based Code and smart growth are associated with potential environmental footprints from an expeditious and thorough exploration of what-if scenarios of the multiple development schemes [18]. Tools of parametric messing, building information modelling (BIM) and virtual reality have been used in Form-Based Code in the former parametric Form-Based Code analysis [13,15,16,18].

Second is combing parametric Form-Based Code with local governance and policies. For example, Kan, a researcher from the University of Hong Kong, believed that Hong Kong government should implement Form-Based Code in the current planning system [19]. According to the findings of Kan, Schnabel, Zhang and Aydin tried to combine parametric tools with the Form-Based Code of Hong Kong to enhance the conventional Form-Based Code for density cities [13]. They argued that parametric Form-Based Code had the capability to improve the management of urban development towards a efficiency administrative system [13]. Sarah’s research in 2020 presented that Form-Based Code could be a potential mechanism for implementing human-centered values in actual built environments, because the professional perspectives that planners and government officials have on urban space were often very different from the lived experiences of the people who actually live in communities [20]. In 2022, Ghosh and Byahut argued the Form-Based Code of 2012 Plan Cincinnati, Ohio, USA had several success measures in terms of citizen engagement in formulating its vision statement and plan making, its success in instigating redevelopment projects to revitalize its dilapidated inner-city neighborhoods, and adoption of Form-Based Codes to encourage place-making ideas and strengthen traditional neighborhoods [21].

Third is comparing parametric Form-Based Code with conventional Form-Based Code. Conventional Form-Based Code describes urban planning by using illustrations and graphs. The planning file covers building function, building configuration, lot occupation, building disposition, and setbacks [22]. It is argued that the regulations of conventional Form-Based Code are not convenient to modify as form-related factors commonly cooperate together to build a specific form [23,24]. To address this issue, parametric models are embedded into Form-Based Code with exported renders and plans [16]. Parametric Form-Based Code has been proved work well to provide flexible layouts and edible components in urban planning [25]. As Schnabel indicated, the use of parametric tools could help enhance the planning process and layouts [13].

However, little research has fully examined the extent to which parametric Form-Based Code integrates design criteria of environmental health and sustainability. There is merely mature standardized evaluation framework to examine if parametric Form-Based Code support urban communities’ sustainable development. Sustainable development in communities takes into account, and addresses, multiple human needs. It should be a place where people can access to green buildings, economic opportunities, a safe and healthy space to communicate within neighborhoods, and a sense of community [26]. Sustainability is of central importance for implementing Form-Based Code in planning [23]. Whilst a Form-Based Code can reflect the morphology and intentions of planning through parametric software, the codes need to be examined by sustainability-related principles for real practice.

## 3. Methods

LEED-ND works as the major method to examine parametric Form-Based Code as it is considered useful in creating sustainable urban morphology [27,28]. Scholars such as Garde, Kim, and Tsai used LEED-ND to evaluate Miami’s Form-Based Code [1]. It is proved helpful in examining Form-Based Code generally, but has not been tested in parametric Form-Based Code with multiple layers and complex transect types. This research innovatively extends LEED-ND’s application to evaluate if parametric Form-Based Code could support sustainable development of communities and neighborhoods. 

**Figure 1:**
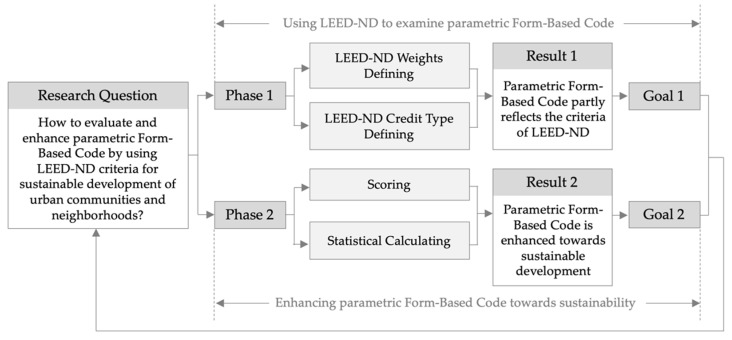
Methodological framework.

The methodological framework consists of two phases (Figure 1). Phase one comprises LEED-ND weights and credit type defining. LEED-ND system contains multiple principle categories. Each category consists of different credit types. The definition before evaluating parametric Form-Based Code is necessary to limit the scope of weights and credits. Phase one targets to achieve the first research goal that analyzing if parametric Form-Based Code reflects the criteria of LEED-ND. Phase two includes scoring and statistical calculating. The method of five-point scale works to measure the concordance between rating system and parametric Form-Based Code. The parametric Form-Based Code of Hong Kong acts as an example to test its score by using the five-point scale method. Six communities in central urban area are selected to be examined by LEED-ND. Thus the examination results reflects an average quality of the parametric Form-Based Code. Evaluating index, including weighted concordance score, raw frequencies, normalized frequencies, and maximum concordance scores, are measured through statistical calculating. Phase two targets to achieve the second research goal that enhancing parametric Form-Based Code towards sustainable development in urban communities and neighborhoods.

**Figure 2:**
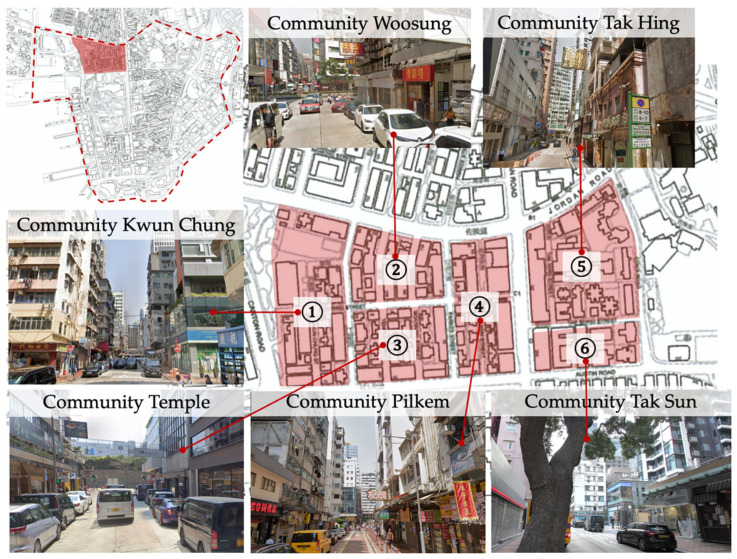
Target communities and streetscapes.

The selected communities, including Community Kwun Chung, Woosung, Temple, Pilkem, Tak Hing, and Tak Sun, locate at Jordan Road Area, Tsim Sha Tsui district of Hong Kong. Figure 2 presents the streetscape of the communities. These communities have very limited land resources, the locals’ works are “an inch of land, an inch of gold”. Choosing the communities of Tsim Sha Tsui is a response to the practical uncertainty about the desirability of parametric Form-Based Code. Mixed land use is a tradition accepted by the society of density cities, so aligns with a parametric Form-Based Code approach to urban planning. While parametric Form-Based Code’s capability to maintain a sustainable development has yet to be examined. The communities of Tsim Sha Tsui could work as the site to evaluate if they are sustainable under parametric Form-Based Code, as well as provide a reference for the rest of Hong Kong and other high-density cities. 

### 3.1. Weights and Credit Types Defining

LEED-ND rating system consists of five categories, including smart location & linkage, neighborhood pattern & design, green infrastructure & buildings, innovation, and regional priority. The weighting factors have the specific criteria of each of the five categories. Comprehensively considering the relevance of sustainable development of urban communities, criteria of smart location & linkage, neighborhood pattern & design, and green infrastructure & buildings are chosen for generating the weighting system.

- Smart location & linkage

Criteria of smart location & linkage in LEED-ND seeks “to encourage development within and near existing communities and public transit infrastructure. To encourage improvement and redevelopment of existing cities, suburbs, and towns while limiting the expansion of the development footprint in the region. To reduce vehicle trips and vehicle distance travelled. To reduce the incidence of obesity, heart disease, and hypertension by encouraging daily physical activity associated with walking and bicycling” [29]. It provides the credits for the weighting system to measure dense-development and urban morphology regulation for a built environment.

- Neighborhood pattern & design

Criteria of neighborhood pattern & design in LEED-ND intends “to promote transportation efficiency and reduce vehicle distance travelled. To improve public health by providing safe, appealing, and comfortable street environments that encourage daily physical activity and avoid pedestrian injuries” [29]. It provides credits for the weighting system to evaluate performance regulation on a community-scale.

- Green infrastructure & buildings

Criteria of green infrastructure & buildings in LEED-ND is applied “to encourage the design, construction, and retrofit of buildings using green building practices” [29]. Because parametric Form-Based Code contains building form standards, it is appropriate to include the criteria of green infrastructure & buildings in the weighting system. 

Innovation criteria encourage projects to achieve exceptional or innovative performance [29]. Regional priority criteria provide an incentive for the achievement of credits that address geographically specific environmental, social equity, and public health priorities [29]. Innovation and regional priority criteria are not appropriate for this weighting structure. These two criteria are basically descriptive without weights that can be scored. Thus they are not selected in the following quantitative analysis of scoring and statistical calculating. There are different criteria types in each weights. For instance, smart location & linkage includes smart location & linkage prerequisite, location & transportation credit, and smart location & linkage credit. The weighting system limits the criteria as credit types as they are quantized by explicit values to score.

### 3.2. Scoring and Statistical Calculating

In this research, parametric Form-Based Code is scored on a five-point scale to measure its concordance with the LEED-ND rating system. This grading approach is aligned with Garde’s research in 2015. As Garde stated, that work relied primarily on the weighted concordance score (W) of LEED-ND criteria reflected in the codes to interpret the results and use thresholds to infer the strength of these scores [1]. If the value of W is equal with or higher than 2.5, the LEED-ND principles are strongly incarnated in parametric Form-Based Code. If W is between 1 and 2.5, it indicates the parametric Form-Based Code contains the LEED-ND principles moderately. If W is equal with or lower than 1, the parametric Form-Based Code rarely meets the LEED-ND criterion.

Four data categories comprise the measurement system. They are raw frequencies (F), normalized frequencies (N), maximum concordance scores (M), and weighted concordance scores (W) of LEED-ND criteria reflected in codes [1]. F is a combination of credits. For example, in the section on smart location & linkage credit: preferred locations, the requirements have three options. They are Location Type (1–5 points), Connectivity (1–5 points) and Designed High-Priority Locations (3 points). F equals the value of the sum of the points parametric Form-Based Code achieves in each option. The total value may be up to 10 points according to the LEED-ND rating system regulations. F reflects the normalized raw frequencies. Calculating F standardizes raw frequencies into the closed interval [0, 1]. According to the values of F, N is calculated as:

(1) $$N=\frac{F-F_{\min}}{F_{\max}-F_{\min}}$$

where:

N is the normalised raw frequencies,

F is the raw frequencies,

F_min_ is the minimum F in the code of specific zone type, and

F_max_ is the maximum F in the code of specific zone type.

M grades the parametric Form-Based Code’s level of concordance with LEED-ND criteria. This research uses the measurement manners proposed by Garde in 2015 [1]. Table 1 describes the concordance levels and measurement descriptions. 

W is calculated as:

(2) $$W=\frac{M_{1}*W_{\mathrm{t1}}+M_{2}*W_{\mathrm{t2}}+\cdots +M_{n}*W_{\mathrm{tn}}}{W_{\mathrm{t1}}+W_{\mathrm{t2}}+\cdots +W_{\mathrm{tn}}}$$

where:

W is the weighted concordance value,

M_1_ is the concordance value of sub-criterion 1,

W_t1_ is the weight for sub-criterion 1,

M_2_ is the concordance value of sub-criterion 2,

W_t2_ is the weight for sub-criterion 2,

M_n_ is the concordance value of sub-criterion n, and

W_tn_ is the weight for sub-criterion n.

The weight of each sub-criterion is based on the maximum credits parametric Form-Based Code can achieve in each sub-criterion and the maximum credits of each criterion. It is calculated as:

(3) $$W_{\mathrm{tx}}=\frac{S_{\mathrm{maxx}}*T}{S_{\mathrm{max1}}+S_{\mathrm{max2}}+\cdots +S_{\mathrm{maxn}}}$$

where: 

W_tx_ is the weight for sub-criterion x,

S_maxx_ is the maximum score that sub-criterion x can offer,

S_max1_ is the maximum score that sub-criterion 1 can offer,

S_max2_ is the maximum score that sub-criterion 2 can offer,

S_maxn_ is the maximum score that each sub-criterion can offer, and

T is the top point that each criterion can offer.

## 4. Results

The scoring results are assessed from the perspectives of smart location & linkage, neighborhood pattern & design, and green infrastructure & buildings. This section describes further details about the findings to explore the sustainability of parametric Form-Based Code through evaluating the level of concordance between LEED-ND and the Form-Based Code. 

The evaluation results can be expressed quantitatively. For example, the preferred locations criterion of smart location & linkage has three sub-criteria, including Option 1: Location Type (1–5 points), Option 2: Connectivity (1–5 points) and Option 3: Designed High-Priority Locations (3 points). LEED-ND regulates that the highest point of Option 1 is 5, Option 2 is 5 and Option 3 is 3. The total score is out of 10. Using the equation (3), the weight of Option 1 is 3.85, the weight of Option 2 is 3.85, and the weight of Option 3 is 2.30. Assuming the specific code’s concordance value of Option 1 is 2 (Fair), the value of Option 2 is 2 (Fair) and the value of Option 3 is 1 (weak), the specific code’s W is 1.8 calculated by equation (2) and F is 5 by adding the score of each option together. The W value of 1.8 is between 1 and 2.5, which indicates that the specific code meets the LEED-ND principles moderately. According to this manner of measurement, Table 2, Table 3 and Table 4 show the scores of smart location & linkage, neighborhood pattern & design, and green infrastructure & buildings scores of F, N, M, and W for the parametric Form-Based Code.

The scoring results provide a reference for a Form-Based Code team to measure the concordance between LEED-ND and parametric Form-Based Code. This is depicted in Table 5 according to the data in above tables. The light shading area indicates that concordance values are less than or equal with 1, which means nearly no principle of LEED-ND is reflected in the parametric Form-Based Code. The medium shading area with one “X” mark indicates that concordance values are between 1 and 2.5, which means that part of the principles of LEED-ND are reflected in the code; the code moderately aligns with LEED-ND criteria. The dark shading area with two “X” marks means the concordance values are equal to, or higher than 2 and the principles of LEED-ND are strongly reflected in the parametric Form-Based Code.

## 5. Discussion

### 5.1. Smart Location & Linkage

Generally, the measurement results indicate that the parametric Form-Based Code is not fully aligned with the criteria of smart location & linkage of LEED-ND. There are seven weights in the category of smart location & linkage. Only the principles of Preferred Locations are moderately reflected. The purpose of this weight is to encourage new development in existing cities, suburbs, and towns. It is apparent in the parametric Form-Based Code’s intention because of the plenty of old communities and compact space in urban areas. Weights such as Brownfield Remediation and Water Body Conservation, are not related to parametric Form-Based Code. Others, like Housing and Jobs Proximity and Steep Slope Protection, are partly included in parametric Form-Based Code but do not meet the middle-rank standard. 

From the sustainable development of urban communities and neighborhoods, parametric Form-Based Code weakly accords with LEED-ND standards. Some LEED-ND standards has been applied to examine parametric Form-Based Code while rare standards are offered specifically for form-related regulations. As the former review argued, Form-Based Code fosters predictable built results and a quality public realm [3]. Thus natural resource protection is involved to limit the edges of built environment planned with parametric Form-Based Code. Parametric Form-Based Code should add regulations, including conservation of habitat or wetlands and water bodies, protection of natural topography, and housing and jobs proximity, to achieve a healthy socio-environment and sustainable human-natural habitat.

### 5.2. Neighborhood Pattern & Design

Criteria of neighborhood patter & design contain fourteen weights for measuring transportation connection, public health and comfortable street environment. The weights of Transit Facilities, Transportation Demand Management, and Access to Civic and Public Space are strongly reflected in the parametric Form-Based Code as the Form-Based Code regulates the transit waiting areas to be safe, convenient and comfortable. Multi-mode travel is encouraged and ranges from the public subway to walking. Public space can be organized as connecting work and home at different levels to fulfil the requirement of “non-residential use entrances within a 1/4 mile (400 m) walk of at least one civic and passive use space” [29].

The weights of Compact Development and Mixed-Use Neighborhoods are strongly reflected in the paramedic Form-Based Code. These weights are about conserving land by encouraging the development of existing infrastructure and car-free, mixed-use communities. It is aligned with the former review that parametric Form-Based Code provides flexible layouts and edible components in urban planning [25]. Compact and mixed-use are encouraged because of the flexible feature of parametric Form-Based Code. The weights of Connected and Open Community are strongly consistent with the regulations of community 3–6 and moderately reflected in community 1 and 2. Community 1 to 3 partly achieve the weight of Walkable Streets, while community 4 to 6 strongly reflect these weights. Other weights like Local Food Production, Neighborhood Schools or Visitability and Universal Design are not related to the Form-Based Code, or are partly reflected but cannot meet the moderate standard.

Nearly half of the neighborhood pattern & design criteria are moderately reflected in the parametric Form-Based Code. The value of M in each community indicates that criterion is addressed in the regulations to more than a minimum extent, but still not to the degree of achieving the maximum possible LEED-ND points. Although the concordance values are still lower than 2.5, they consistently meet the moderate standard of the neighborhood pattern & design weights of LEED-ND. Parametric Form-Based Code could be enhanced by adding regulations in the aspects of green streets, neighborhood schools and community involvement to create child friendly community and quality built environment.

### 5.3. Green Infrastructure & Buildings

There are seventeen weights in the category of green infrastructure & buildings. Only two weights, Historic Resource Preservation and Adaptive Reuse and Minimized Site Disturbance, can be strongly reflected in the parametric Form-Based Code and one weight, Building Reuse, is moderately reflected. Other weights, such as Certified Green Building, Minimum Building Energy Performance, and Indoor Water Use Reduction are outside the scope of parametric Form-Based Code. To create sustainable communities and neighborhoods in urban areas, parametric Form-Based Code should involve the codes of green buildings’ construction or reconstruction. According to Santamouris and Vasilakopoulou, building energy consumption accounts for a high proportion in cities [30]. The built environment is not just a collection of buildings though, but the physical expression and manifestation of numerous economic, social, and environmental process strongly related to the human activities and the changing needs of society [31]. 

In summary, a small number of the criteria of smart location & linkage, neighborhood patter & design, and green infrastructure & buildings of LEED-ND are concordant with parametric Form-Based Code. The criteria of neighborhood pattern & design are more moderately or strongly reflected. On average, parametric Form-Based Code has a weak concordance with LEED-ND criteria. To enhance parametric Form-Based Code towards a sustainable development in urban communities and neighborhoods, parametric Form-Based Code needs to involve regulations related to natural resource conservation, green space and energy-efficient buildings. 

### 5.4. Proposed Enhancements on Parametric Form-Based Code

According to the LEED-ND examination results, it could be summarized that three strategies may support the enhancement of parametric Form-Based Code. First is adding regulations of conservation of habitat or wetlands and water bodies, protection of natural topography, and housing and jobs proximity. Urban communities and neighborhoods tend to balance land usage, transportation congestion and population growth. Natural and societal resources are both essential to compose a sustainable socio-environment. Second is involving green streets, neighborhood schools and community involvement to create child friendly community and quality built environment. The scoring result of neighborhood pattern & design reflects that streets with green canopy, nearby schools and community involvement are important weights to a sustainable development. Parametric Form-Based Code thus should emphasizes site design and building form to fit specific places by fulfilling local requirements. Third is bringing green buildings’ construction or reconstruction into the regulation system. This is align with Natanian, Aleksandrowicz, and Auer’s research that promoting green residential and office buildings should help designers and policy makers contextualize nearly zero energy community concepts as well as define new criteria and goals [32]. This research limits the target sites in Tsim Sha Tsui, Hong Kong. Findings are mainly from the analysis of parametric Form-Based Code of communities and neighborhoods of Jordan Road Area. Further studies in different cases could be conducted with more experiments and examinations. 

## 6. Conclusions

This research highlights the evaluation system of parametric Form-Based Code by LEED-ND methods, with a view towards healthy socio-environment and sustainable development of urban communities and neighborhoods. Quantitative data analysis were conducted on parametric Form-Based Code by using multiple weights and scores of LEED-ND. Results indicate that parametric Form-Based Code has relatively weak concordance with the criteria of smart location & linkage and green infrastructure & buildings, and strong concordance with the criteria of neighborhood pattern & design. A lack of regulations about natural resource conservation, green space and energy-efficient buildings diminish the sustainability of parametric Form-Based Code. According to the above analysis of code shortages, three strategies for enhancing parametric Form-Based Code are proposed, including adding conservation regulations, involving green streets, neighborhood schools and community involvement and encouraging green buildings. These strategies support the sustainable development of urban communities and neighborhoods according to LEED-ND standards. Extending the application of parametric Form-Based Code towards sustainable development of density cities should be further studied in future.

## Figures and Tables

**Table 1:** Concordance levels and measurement descriptions *.

Concordance Score	Concordance Level	Concordance Measurement
4	Excellent	Criterion is addressed in parametric Form-Based Code to the extent that maximum LEED-ND points can be achieved
3	Good	Criterion is addressed in parametric Form-Based Code to the extent that more than minimum but less than maximum LEED-ND points can be achieved
2	Fair	Criterion is addressed in parametric Form-Based Code but no LEED-ND points can be achieved
1	Weak	Criterion is addressed in parametric Form-Based Code but no LEED-ND points can be achieved
0	None	LEED-ND criterion not addressed or has no relevance codes

* The concordance measurement descriptions are following Garde’s evaluation manners in 2015.

**Table 2:** Scoring results of smart location & linkage.

Community No.	Value Types	Values								
		Preferred Locations (10)	Brownfield Remediation (2)	Housing and Jobs Proximity (3)	Steep Slope Protection (1)	Site Design for Habitat or Wetland and Water Body Conservation (1)	Restoration of Habitat or Wetland and Water Bodies (1)	Long-Term Conservation Management of Habitat or Wetlands and Water Bodies (1)	Average	Subtotal
1	F	8.0	0.0	2.0	0.0	0.0	0.0	0.0	**1.4**	**10.0**
	N	1.0	0.0	0.3	0.0	0.0	0.0	0.0	**0.2**	**1.3**
	M	4.0	0.0	3.0	0.0	0.0	0.0	0.0	**1.0**	**-**
	W	2.3	0.0	1.0	0.0	0.0	0.0	0.0	**0.5**	**-**
2	F	8.0	0.0	2.0	0.0	0.0	0.0	0.0	**1.4**	**10.0**
	N	1.0	0.0	0.3	0.0	0.0	0.0	0.0	**0.2**	**1.3**
	M	4.0	0.0	2.0	0.0	0.0	0.0	0.0	**0.9**	**-**
	W	2.3	0.0	0.7	0.0	0.0	0.0	0.0	**0.4**	**-**
3	F	7.0	0.0	1.0	0.0	0.0	0.0	0.0	**1.1**	**8.0**
	N	1.0	0.0	0.1	0.0	0.0	0.0	0.0	**0.2**	**1.1**
	M	4.0	0.0	2.0	0.0	0.0	0.0	0.0	**0.9**	**-**
	W	2.3	0.0	0.7	0.0	0.0	0.0	0.0	**0.4**	**-**
4	F	7.0	0.0	1.0	0.0	0.0	0.0	0.0	**1.1**	**8.0**
	N	1.0	0.0	0.1	0.0	0.0	0.0	0.0	**0.2**	**1.1**
	M	4.0	0.0	2.0	0.0	0.0	0.0	0.0	**0.9**	**-**
	W	2.3	0.0	0.7	0.0	0.0	0.0	0.0	**0.4**	**-**
5	F	7.0	0.0	0.0	0.0	0.0	0.0	0.0	**1.0**	**7.0**
	N	1.0	0.0	0.0	0.0	0.0	0.0	0.0	**0.2**	**1.0**
	M	4.0	0.0	2.0	0.0	0.0	0.0	0.0	**0.9**	**-**
	W	2.3	0.0	0.7	0.0	0.0	0.0	0.0	**0.4**	**-**
6	F	7.0	0.0	0.0	0.0	0.0	0.0	0.0	**1.0**	**7.0**
	N	1.0	0.0	0.0	0.0	0.0	0.0	0.0	**0.2**	**1.0**
	M	4.0	0.0	2.0	0.0	0.0	0.0	0.0	**0.9**	**-**
	W	2.3	0.0	0.7	0.0	0.0	0.0	0.0	**0.4**	**-**

**Table 3:** Scoring results of neighborhood pattern & design.

Community No.	Value Types	Values															
		Walkable Streets (9)	Compact Development (6)	Mixed-Use Neighborhoods (4)	Housing Types and Affordability (7)	Connected and Open Community (2)	Transit Facilities (1)	Transportation Demand Management (2)	Access to Civic and Public Space (1)	Access to Recreation Facilities (1)	Visit Ability and Universal Design (1)	Community Outreach and Involvement (2)	Local Food Production (1)	Tree-Lined and Shaded Streetscapes (2)	Neighborhood Schools (1)	Average	Subtotal
1	F	3.0	6.0	2.0	2.0	1.0	1.0	2.0	1.0	1.0	0.0	0.0	0.0	2.0	1.0	**1.6**	**22.0**
	N	0.5	1.0	0.3	0.3	0.2	0.2	0.3	0.2	0.2	0.0	0.0	0.0	0.3	0.2	**0.3**	**3.7**
	M	4.0	4.0	4.0	2.0	4.0	3.0	4.0	4.0	4.0	0.0	0.0	0.0	2.0	1.0	**2.6**	**-**
	W	1.3	4.0	4.0	0.9	2.0	3.0	4.0	4.0	4.0	0.0	0.0	0.0	1.0	1.0	**2.1**	**-**
2	F	4.0	6.0	3.0	2.0	1.0	1.0	2.0	1.0	1.0	0.0	0.0	0.0	2.0	1.0	**1.7**	**24.0**
	N	0.7	1	0.5	0.3	0.2	0.2	0.3	0.2	0.2	0.0	0.0	0.0	0.3	0.2	**0.3**	**4.1**
	M	4.0	4.0	4.0	2.0	4.0	3.0	4.0	4.0	4.0	0.0	0.0	0.0	2.0	1.0	**2.6**	**-**
	W	1.8	4.0	4.0	0.9	2.0	3.0	4.0	4.0	4.0	0.0	0.0	0.0	1.0	1.0	**2.1**	**-**
3	F	5.0	6.0	3.0	2.0	2.0	1.0	2.0	1.0	1.0	0.0	0.0	0.0	2.0	1.0	**1.9**	**26.0**
	N	0.8	1.0	0.5	0.3	0.3	0.2	0.3	0.2	0.2	0.0	0.0	0.0	0.3	0.2	**0.3**	**4.3**
	M	4.0	4.0	4.0	2.0	4.0	3.0	4.0	4.0	4.0	0.0	0.0	0.0	2.0	1.0	**2.6**	**-**
	W	2.2	4.0	4.0	0.9	4.0	3.0	4.0	4.0	4.0	0.0	0.0	0.0	1.0	1.0	**2.3**	**-**
4	F	6.0	6.0	4.0	2.0	2.0	1.0	2.0	1.0	1.0	0.0	0.0	0.0	1.0	1.0	**1.9**	**27.0**
	N	1.0	1.0	0.7	0.3	0.3	0.2	0.3	0.2	0.2	0.0	0.0	0.0	0.2	0.2	**0.3**	**4.6**
	M	4.0	4.0	4.0	2.0	4.0	3	4.0	4.0	4.0	0.0	0.0	0.0	2.0	1.0	**2.6**	**-**
	W	3.1	4.0	4.0	0.9	4.0	3.0	4.0	4.0	4.0	0.0	0.0	0.0	1.0	1.0	**2.4**	**-**
5	F	7.0	6.0	3.0	2.0	2.0	1.0	2	1.0	1.0	0.0	0.0	0.0	1.0	1.0	**1.9**	**27.0**
	N	1.0	0.9	0.4	0.3	0.3	0.1	0.3	0.1	0.1	0.0	0.0	0.0	0.1	0.2	**0.3**	**3.7**
	M	4.0	4.0	4.0	2.0	4.0	3.0	4.0	4.0	4.0	0.0	0.0	0.0	2.0	1.0	**2.6**	**-**
	W	3.1	4.0	4.0	0.9	4.0	3.0	4.0	4.0	4.0	0.0	0.0	0.0	2.0	1.0	**2.4**	**-**
6	F	7.0	6.0	3.0	2.0	1.0	1.0	2.0	1.0	1.0	0.0	0.0	0.0	2.0	1.0	**1.9**	**26.0**
	N	1.0	0.9	0.4	0.3	0.1	0.1	0.3	0.1	0.1	0.0	0.0	0.0	0.1	0.1	**0.3**	**3.5**
	M	4.0	4.0	4.0	2.0	4.0	3.0	4.0	4.0	4.0	0.0	0.0	0.0	1.0	1.0	**2.6**	**-**
	W	3.1	4.0	4.0	0.9	4.0	3.0	4.0	4.0	4.0	0.0	0.0	0.0	1.0	1.0	**2.4**	**-**

**Table 4:** Scoring results of green infrastructure & buildings.

Community No.	Value Types	Values																		
		Certified Green Buildings (5)	Optimize Building Energy Performance (2)	Indoor Water Use Reduction (1)	Outdoor Water Use Reduction (2)	Building Reuse (1)	Historic Resource Preservation and Adaptive Reuse (2)	Minimized Site Disturbance (1)	Rainwater Management (4)	Heat Island Reduction (1)	Solar Orientation (1)	Renewable Energy Production (3)	District Heating and Cooling	Infrastructure Energy Efficiency (1)	Wastewater Management (2)	Recycled and Reused Infrastructure (1)	Solid Waste Management (1)	Light Pollution Reduction (1)	Average	Subtotal
1	F	0.0	0.0	0.0	0.0	1.0	2.0	1.0	0.0	0.0	0.0	0.0	0.0	0.0	0.0	0.0	0.0	0.0	**0.2**	**4**
	N	0.0	0.0	0.0	0.0	0.5	1.0	0.5	0.0	0.0	0.0	0.0	0.0	0.0	0.0	0.0	0.0	0.0	**0.1**	**2**
	M	0.0	0.0	0.0	0.0	2.0	3.0	3.0	0.0	0.0	0.0	0.0	0.0	0.0	0.0	0.0	0.0	0.0	**0.5**	**-**
	W	0.0	0.0	0.0	0.0	2.0	3.0	3.0	0.0	0.0	0.0	0.0	0.0	0.0	0.0	0.0	0.0	0.0	**0.5**	**-**
2	F	0.0	0.0	0.0	0.0	1.0	2.0	1.0	0.0	0.0	0.0	0.0	0.0	0.0	0.0	0.0	0.0	0.0	**0.2**	**4**
	N	0.0	0.0	0.0	0.0	0.5	1.0	0.5	0.0	0.0	0.0	0.0	0.0	0.0	0.0	0.0	0.0	0.0	**0.1**	**2**
	M	0.0	0.0	0.0	0.0	2.0	3.0	3.0	0.0	0.0	0.0	0.0	0.0	0.0	0.0	0.0	0.0	0.0	**0.5**	**-**
	W	0.0	0.0	0.0	0.0	2.0	3.0	3.0	0.0	0.0	0.0	0.0	0.0	0.0	0.0	0.0	0.0	0.0	**0.5**	**-**
3	F	0.0	0.0	0.0	0.0	1.0	2.0	1.0	0.0	0.0	0.0	0.0	0.0	0.0	0.0	0.0	0.0	0.0	**0.2**	**4**
	N	0.0	0.0	0.0	0.0	0.5	1.0	0.5	0.0	0.0	0.0	0.0	0.0	0.0	0.0	0.0	0.0	0.0	**0.1**	**2**
	M	0.0	0.0	0.0	0.0	2.0	3.0	3.0	0.0	0.0	0.0	0.0	0.0	0.0	0.0	0.0	0.0	0.0	**0.5**	**-**
	W	0.0	0.0	0.0	0.0	2.0	3.0	3.0	0.0	0.0	0.0	0.0	0.0	0.0	0.0	0.0	0.0	0.0	**0.5**	**-**
4	F	0.0	0.0	0.0	0.0	1.0	2.0	1.0	0.0	0.0	0.0	0.0	0.0	0.0	0.0	0.0	0.0	0.0	**0.2**	**4**
	N	0.0	0.0	0.0	0.0	0.5	1.0	0.5	0.0	0.0	0.0	0.0	0.0	0.0	0.0	0.0	0.0	0.0	**0.1**	**2**
	M	0.0	0.0	0.0	0.0	2.0	3.0	3.0	0.0	0.0	0.0	0.0	0.0	0.0	0.0	0.0	0.0	0.0	**0.5**	**-**
	W	0.0	0.0	0.0	0.0	2.0	3.0	3.0	0.0	0.0	0.0	0.0	0.0	0.0	0.0	0.0	0.0	0.0	**0.5**	**-**
5	F	0.0	0.0	0.0	0.0	1.0	2.0	1.0	0.0	0.0	0.0	0.0	0.0	0.0	0.0	0.0	0.0	0.0	**0.2**	**4**
	N	0.0	0.0	0.0	0.0	0.5	1.0	0.5	0.0	0.0	0.0	0.0	0.0	0.0	0.0	0.0	0.0	0.0	**0.1**	**2**
	M	0.0	0.0	0.0	0.0	2.0	3.0	3.0	0.0	0.0	0.0	0.0	0.0	0.0	0.0	0.0	0.0	0.0	**0.5**	**-**
	W	0.0	0.0	0.0	0.0	2.0	3.0	3.0	0.0	0.0	0.0	0.0	0.0	0.0	0.0	0.0	0.0	0.0	**0.5**	**-**
6	F	0.0	0.0	0.0	0.0	1.0	2.0	1.0	0.0	0.0	0.0	0.0	0.0	0.0	0.0	0.0	0.0	0.0	**0.2**	**4**
	N	0.0	0.0	0.0	0.0	0.5	1.0	0.5	0.0	0.0	0.0	0.0	0.0	0.0	0.0	0.0	0.0	0.0	**0.1**	**2**
	M	0.0	0.0	0.0	0.0	2.0	3.0	3.0	0.0	0.0	0.0	0.0	0.0	0.0	0.0	0.0	0.0	0.0	**0.5**	**-**
	W	0.0	0.0	0.0	0.0	2.0	3.0	3.0	0.0	0.0	0.0	0.0	0.0	0.0	0.0	0.0	0.0	0.0	**0.5**	**-**

**Table 5:** Concordance between LEED-ND and parametric Form-Based Code.

LEED-ND Criteria	Community No.					
	1	2	3	4	5	6
Smart location & linkage						
General						
Preferred Locations (10)	X	X	X	X	X	X
Brownfield Remediation (2)						
Housing and Jobs Proximity (3)						
Steep Slope Protection (1)						
Site Design for Habitat or Wetland and Water Body Conservation (1)						
Restoration of Habitat or Wetland and Water Bodies (1)						
Long-Term Conservation Management of Habitat or Wetlands and Water Bodies (1)						
Neighborhood pattern & design						
General	X	X	X	X	X	X
Walkable Streets (9)	X	X	X	XX	XX	XX
Compact Development (6)	XX	XX	XX	XX	XX	XX
Mixed-Use Neighborhoods (4)	XX	XX	XX	XX	XX	XX
Housing Types and Affordability (7)						
Connected and Open Community (2)	X	X	XX	XX	XX	XX
Transit Facilities (1)	XX	XX	XX	XX	XX	XX
Transportation Demand Management (2)	XX	XX	XX	XX	XX	XX
Access to Civic and Public Space (1)	XX	XX	XX	XX	XX	XX
Access to Recreation Facilities (1)	XX	XX	XX	XX	XX	XX
Visitability and Universal Design (1)						
Community Outreach and Involvement (2)						
Local Food Production (1)						
Tree-Lined and Shaded Streetscape (2)						
Neighborhood Schools (1)						
Green infrastructure & buildings						
General						
Certified Green Buildings (5)						
Optimize Building Energy Performance (2)						
Indoor Water Use Reduction (1)						
Outdoor Water Use Reduction (2)						
Building Reuse (1)	X	X	X	X	X	X
Historic Resource Preservation and Adaptive Reuse (2)	XX	XX	XX	XX	XX	XX
Minimized Site Disturbance (1)	XX	XX	XX	XX	XX	XX
Rainwater Management (4)						
Heat Island Reduction (1)						
Solar Orientation (1)						
Renewable Energy Production (3)						
District Heating and Cooling (2)						
Infrastructure Energy Efficiency (1)						
Wastewater Management (2)						
Recycled and Reused Infrastructure (1)						
Solid Waste Management (1)						
Light Pollution Reduction (1)						
